# Rare gut microbiota associated with breeding success, hormone metabolites and ovarian cycle phase in the critically endangered eastern black rhino

**DOI:** 10.1186/s40168-019-0639-0

**Published:** 2019-02-15

**Authors:** Rachael E. Antwis, Katie L. Edwards, Bryony Unwin, Susan L. Walker, Susanne Shultz

**Affiliations:** 1 School of Environment and Life Sciences, University of Salford, Salford, UK; 2 Center for Species Survival, Smithsonian Conservation Biology Institute, Front Royal, VA USA; 3 North of England Zoological Society, Chester Zoo, Upton-by-Chester, UK; 4 School of Earth and Environmental Sciences, University of Manchester, Manchester, UK

**Keywords:** Progestagens, Glucocorticoids, Faecal metabolites, Conservation breeding programmes, Indicator analysis

## Abstract

**Background:**

Host microbiomes play a role in hormone production and subsequent fertility in humans, but this is less well understood in non-model organisms. This is of particular relevance to species in zoo-based conservation breeding programmes, as relationships between host microbiome composition and reproductive output may allow for the development of microbial augmentation strategies to improve success. Here, we characterise faecal bacterial communities of breeding and non-breeding eastern black rhino (*Diceros bicornis michaeli*) using 16S rRNA gene amplicon sequencing and quantify progestagen and glucocorticoid metabolite concentrations through enzyme immunoassays to identify such relationships.

**Results:**

We identified significant differences in black rhino gut microbiome composition according to ID, institution, breeding success and ovarian cycle phase. In particular, the gut microbiome during pregnancy and post-parturition was significantly altered. Around a third of bacterial genera showed more than ± 10% correlation with either progestagen and/or glucocorticoid concentration, and in general, microbial genera correlated with both hormones in the same direction. Through a combination of analyses, we identified four genera (*Aerococcaceae*, *Atopostipes*, *Carnobacteriaceae * and *Solobacterium*) that were significantly associated with breeding success, pregnancy and/or post-parturition, and higher faecal progestagen metabolite concentrations. These genera had a lower-than-average relative abundance in the gut microbiome.

**Conclusion:**

Our results indicate that many members of the gut microbiome of black rhino are associated with hormone production and breeding success, and some members of the rare microbiota appear to be particularly important. Although the directionality of the relationship is unclear, the variation in gut microbiome communities represents a potential biomarker of reproductive health. We identified four genera that were associated with multiple indicators of reproductive output; these could be candidate probiotics to improve the breeding success of black rhino in zoo-based conservation breeding programmes. Further work is required to understand the efficacy and feasibility of this, either directly through microbial augmentation (e.g. probiotics) or indirectly via dietary manipulation or prebiotics.

**Electronic supplementary material:**

The online version of this article (10.1186/s40168-019-0639-0) contains supplementary material, which is available to authorized users.

## Background

Host-associated microbial communities (“microbiomes”) play a critical role in influencing a diverse suite of whole-organism functions, with classic examples including immunity and metabolism [1–4]. Increasingly, the role of microbiomes in regulating hormone and steroid production is being recognised in humans and model organisms. Not only do host hormones shape the structure and function of the host microbiome, but the microbiome can also alter host production and regulation of hormones (e.g. catecholamines, oestrogens, testosterone, thyroid and growth hormones) and alter hormone-associated host gene expression profiles [5–7].

Complex and bidirectional communication between host microbiota and the central nervous system has been identified, with considerable work in humans looking at the gut microbiome in particular [8]. The gut microbiome can influence the central nervous system and vice versa, as the gastrointestinal tract acts as scaffold for the various pathways of the central nervous system [5–7]. Perceived stress induces a chemical response via the hypothalamic–pituitary–adrenal axis in the form of glucocorticoid hormone release (usually cortisol or corticosterone), and it is thought that the bacterial response to such biochemicals results in changes to the gut microbiome [8–12]. The gut microbiota can also produce endocrine molecules including biologically active catecholamines (norepinephrine and dopamine) [13], and the gut microbiota can degrade hormones and change host gene expression, with consequences for reproductive success [14]. Thus, in addition to the well-characterised suite of reproductive and adrenal hormones involved in reproduction, host microbiomes have the potential to affect individual fitness. For example, the human gut microbiome regulates oestrogen production through the secretion of β-glucuronidase enzymes that allow oestrogen to bind to downstream receptors [15], and glucocorticoids can be converted to androgens by specific human gut microbes (e.g. *Clostridium scindens*; [16]). Disruption of the human gut microbiome causes changes in circulating oestrogen, whereas manipulation of the gut microbiome can alter the outcome of oestrogen-related pathologies including infertility [17]. In addition, the microbiome of the reproductive tract can also affect a range of pregnancy outcomes for humans, and research is ongoing to understand how the microbiome could be manipulated to improve pregnancy rates and completion to term [18–20].

Given the importance of host microbiota in human health and reproduction, we have a poor understanding of these relationships in other species. More recently, interactions between host microbiomes and hormone production have been identified in non-human and non-model organisms. Noguera et al. [21] reported the loss of gastrointestinal bacterial taxa in yellow-legged gulls (*Larus michahellis*) with experimentally elevated corticosterone levels. Stothart et al. [22] show that higher faecal glucocorticoid metabolite concentrations are associated with reduced oral bacterial diversity in wild red squirrels (*Tamiasciurus hudsonicus*), and similarly, the abundance of particular bacterial genera was either positively or negatively correlated with faecal glucocorticoid metabolite concentrations in free-ranging western lowland gorillas (*Gorilla gorilla gorilla*) [23]. As with humans, these patterns extend beyond the gut microbiome for other species. Miller et al. [24] identified significant differences in the vaginal microbiome of wild baboons (*Papio cynocephalus*) according to reproductive state (i.e. pregnancy, cycling and postpartum amenorrhea). The authors also showed that the microbiome altered across the ovarian cycle, with a particularly distinct microbiome characterised by high abundance of *Streptococcus*, *Trichococcus*, *Sneathia* and *Bifidobacterium* during ovulation [24]; however, the significance of such changes for reproductive success is not known.

Interactions between host microbiota and hormone profiles are of particular relevance to animals maintained in captive environments, such as zoos and aquaria, which play an important role in ex situ conservation programmes [25–28]. Optimising the health and fitness of captive animals can maximise longevity and reproductive output and thus support sustainable zoo populations. The importance of a “healthy microbiome” for captive animals is increasingly being recognised, although characterising the taxonomic and functional attributes of this is still in its infancy [29–31]. Given the importance of successful breeding in captive collections, it is of interest to identify whether components of the microbiome are associated with reproductively successful individuals, and to characterise the relationships between microbiome composition and hormones involved in reproductive output. This includes reproductive hormones as well as glucocorticoids. Glucocorticoids are more commonly associated with the adrenal stress response and can be related to disruption of reproductive function; for example, higher variability in faecal glucocorticoid metabolite concentration is associated with irregular ovarian cyclicity in captive white rhinos (*Ceratotherium simum*; [32]). However, more recently, glucocorticoids have also been shown to be important for normal ovarian function in a number of zoo animals [33, 34], including the black rhinoceros (K. Edwards, in prep).

Black rhinos (*Diceros bicornis*) are a particularly interesting study system in which to identify relationships between gut microbiota and hormone production. Wild populations are under considerable threat due to poaching for their horns, with approximately 5000 individuals remaining in the wild across a highly fragmented landscape, including only 900 of the eastern black rhino (*D. b. michaeli*) subspecies [35]. Therefore, captive populations are vital to ensure the survival of this species and in the long term, provide individuals for reintroduction [36]. Captive black rhino have, however, suffered historically from low and inconsistent reproductive output caused by irregular ovarian activity and obesity [37, 38]. In addition, temperament differences are associated with higher faecal glucocorticoid metabolite concentrations [37], particularly in nulliparous females. Given the links between microbiome composition, hormone production and reproductive output, identifying components of the microbiome associated with fertility may provide insight into mechanisms that regulate breeding success in this critically endangered species.

Using faecal samples from captive black rhino, we characterised microbiome composition using 16S rRNA amplicon sequencing and identified relationships with glucocorticoid and progestogen metabolites. Specifically, we tested the following hypotheses: (i) differences in microbiome composition are associated with rhino ID, institution, ovarian cycle phase (follicular, luteal, pregnancy or post-parturition) and historical breeding success; (ii) microbiome composition varies according to faecal hormone metabolite concentrations; and (iii) particular microbial taxa are associated with breeding success and ovarian cycle phase.

## Methods

### Study animals, sample collection and faecal hormone metabolite concentrations

We collected data from 16 female eastern black rhinoceros across three UK zoological institutions: Port Lympne Reserve (near Ashford, *n* = 9), Chester Zoo (Chester, *n* = 5), and Howletts Wild Animal Park (near Canterbury, *n* = 2) (Table S1). Each facility also housed at least one adult male. The age of the study individuals ranged from 5 to 40 years (Additional file 1: Table S1). Individuals were categorised according to parity; “non-breeding” individuals had never bred or had not produced a calf within the last 7 years (*n* = 11), whereas “breeding” individuals had produced a calf in the last 7 years, including those pregnant at some point in the study period (*n* = 5) (Additional file 1:: Table S1). The average inter-calving interval of this population is 3.5 years, and so 7 years was chosen as the period to represent parity (i.e. double the period during which a female would ideally have produced a subsequent calf [37]). The lack of breeding in non-parous females had previously been attributed to inconsistent cycling [37].

Four to 12 (median = 8, *N* = 130) faecal samples per individual were chosen from across a 21-month period (January 2010 to September 2011) for which both glucocorticoid and progestagen metabolite concentrations had previously been assessed [37]. Faecal samples provide an indication of circulating hormone concentrations during the period of gut passage [39]. Samples were collected as soon as possible after defaecation and frozen at − 20 °C, then shipped to Chester Zoo (UK) for analysis. Faecal glucocorticoid and progestagen metabolites were extracted from homogenised samples using methanol and quantified using enzyme immunoassays as described in Edwards et al. [37].

### DNA extraction and 16S rRNA gene amplicon sequencing

Samples were analysed for bacterial composition using 16S rRNA gene amplicon sequencing according to Kozich et al. [40] and Antwis et al. [41]. We extracted DNA using the QIAamp DNA Stool Mini Kit (Qiagen, UK) following the manufacturer’s protocol, with an additional incubation time of 30 min at 95 °C. We included a blank extraction to act as a negative control and a mock community (BEI Resources, USA) as a positive control. DNA was amplified for the 16S rRNA gene (v4 region) using dual-indexed forward and reverse primers according to Kozich et al. [40]. PCRs were run in duplicate using Solis BioDyne 5x HOT FIREPol® Blend Master Mix, 2 μM primers and 1 μl of sample DNA using thermocycling conditions of 95 °C for 15 min; 28 cycles of 95 °C for 20s, 50 °C for 60s, 72 °C for 60s; and a final extension at 72 °C for 10 min. PCR replicates were combined and cleaned using HighPrep™ PCR clean up beads (MagBio, USA) according to the manufacturers’ instructions. Products were quality checked using an Agilent 2200 TapeStation and quantified on a Qubit™ 3.0 Fluorometer. Samples were pooled according to concentration to minimise sequencing bias. 16S rRNA gene amplicon sequencing was conducted using paired-end reads (2 × 250 bp) with v2 chemistry on the Illumina MiSeq platform at the University of Salford.

### Pre-processing of microbiome data

We conducted all analyses in RStudio (v1.0.153) [42] for R (v3.4.1) [43]. We processed 16S rRNA gene amplicon sequences in DADA2 v1.5.0 [44] (see Additional file 2). A total of 3,208,334 raw sequence reads from 112 samples were generated during sequencing. Modal contig length was 253 bp once paired-end reads were merged. DADA2 identified 20 unique sequence variants in the sequenced mock community sample comprising 20 bacterial isolates. We removed sequence variants (SVs) with length > 260 bp (7 out of 6427 SVs; 0.101% of total sequences) along with chimeras and two SVs found in the negative controls. SVs with fewer than 100 reads across all the samples were also removed [45]. Four samples with low read numbers were removed from further analyses, leaving an average of 22,525 SVs per sample (range 13,392–43,710). We assigned taxonomy using the SILVA v128 database [46, 47]. To determine whether sequencing had provided sufficient coverage, we constructed rarefaction curves for each sample according to richness and Shannon diversity using the *calculate_rarefaction_curves* function [48], which indicated good coverage over ~ 10,000 reads (Additional file 1: Figure S1). We exported the final SV table, taxonomy table and sample metadata to the phyloseq package [49] for further analysis. To provide greater taxonomic detail about unidentified SVs, and to stop the removal of these during analyses that agglomerate to a given taxonomic level, we fully annotated the taxonomy table to species level using higher level assignments (e.g. SV3 was named “Family_Prevotellaceae” at the genus and species levels).

### Microbiome composition by institution and reproductive history

We converted the data to relative abundance and produced a series of NMDS plots in phyloseq using the Bray–Curtis distance matrix to visualise microbiome variation according to ID, institution, reproductive success and ovarian cycle phase. We conducted a permutational ANOVA (PERMANOVA; adonis) in the vegan package [50] to determine the proportion of variation attributable to ID, breeding success, ovarian cycle phase and institution. We constructed stacked plots to visualise the taxonomic composition of bacterial communities according to institution, breeding success and ovarian cycle phase. We used indictor analysis to identify bacterial genera with significantly different prevalence between breeding (i.e. those that had calved in the 7 years previous, *n* = 5) and non-breeding (those that had never calved or had calved more than 7 years ago, *n* = 10) rhinos. For this, we agglomerated species to genus level and calculated relative abundance of each taxa, then conducted indicator analysis using the *multipatt* function in the indicspecies package [51]. We repeated the analysis to identify indicator genera associated with either of the two phases of the ovarian cycle (luteal, follicular), pregnancy, or post-parturition.

### Relationships between microbiome composition and faecal hormone metabolite concentrations

We log-transformed faecal progestagen and glucocorticoid metabolite concentrations to obtain normally distributed data. We conducted a correlation analysis between log progestagen and log glucocorticoid metabolite concentrations and plotted these according to ovarian cycle phase. Differences in hormone metabolite concentrations according to breeding success and ovarian cycle phase were analysed using linear mixed models (with ID and institution as random factors) using the *lmer* function in the lme4 package [52]. We agglomerated data to genus level and used the *associate* function in the microbiome package [53] to identify relationships between the relative abundance of microbial genera and log faecal glucocorticoid and progestogen metabolite concentrations. We constructed heatmaps in ggplot2 [54] to visualise taxa with *r* > ± 0.10 for either of the hormones (correlation coefficients can be a more reliable indication of relationships than *p* values when sample sizes are small; [55]).

## Results

### Microbiome composition by ID, institution, reproductive success and ovarian cycle phase

**Fig. 1:**
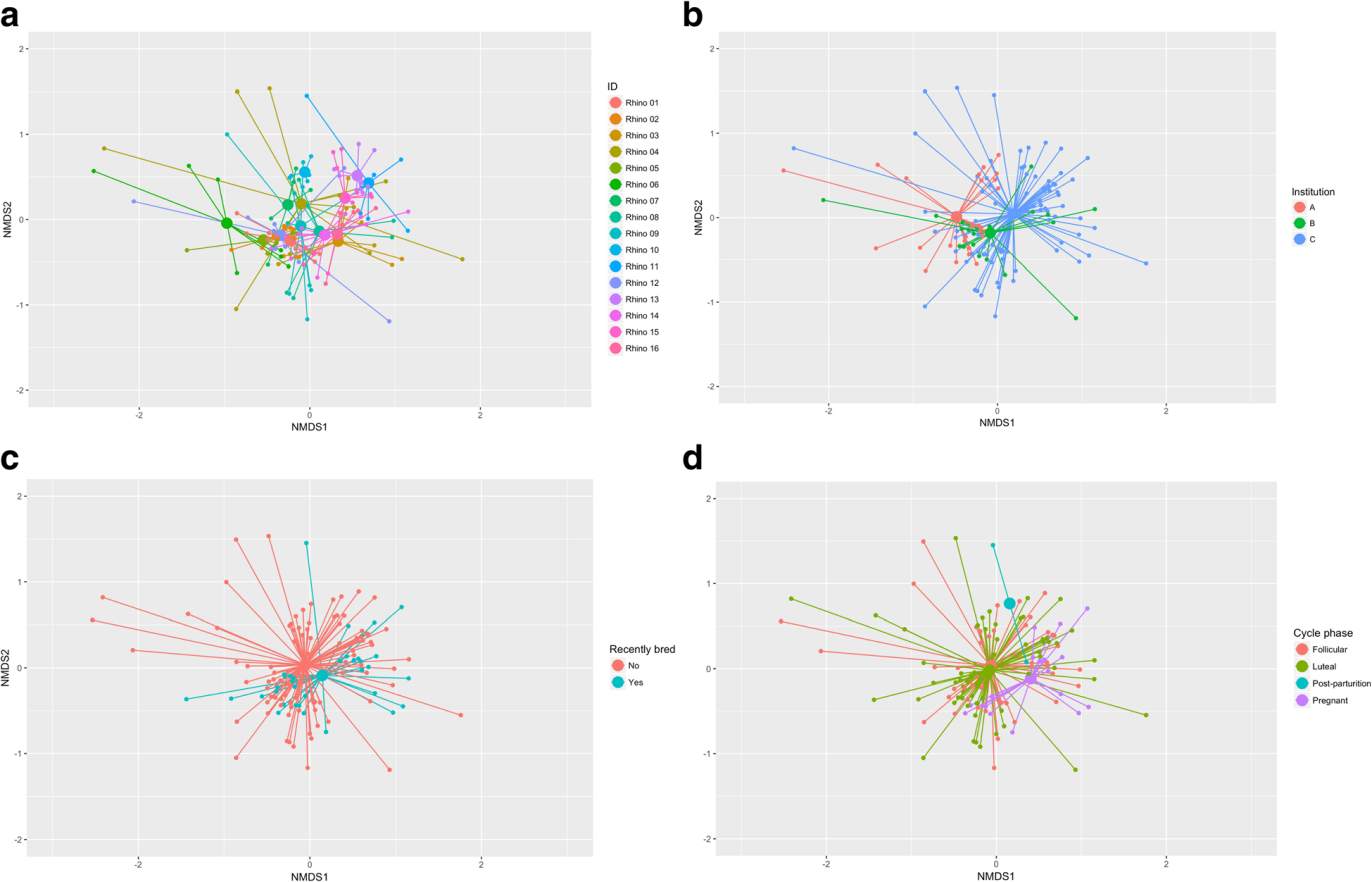
NMDS plots of rhino microbiomes plotted according to **a** ID, **b** institution, **c** reproductive success and **d** cycle phase. Smaller dots indicate individual samples and larger filled circles indicate group centroids

**Fig. 2:**
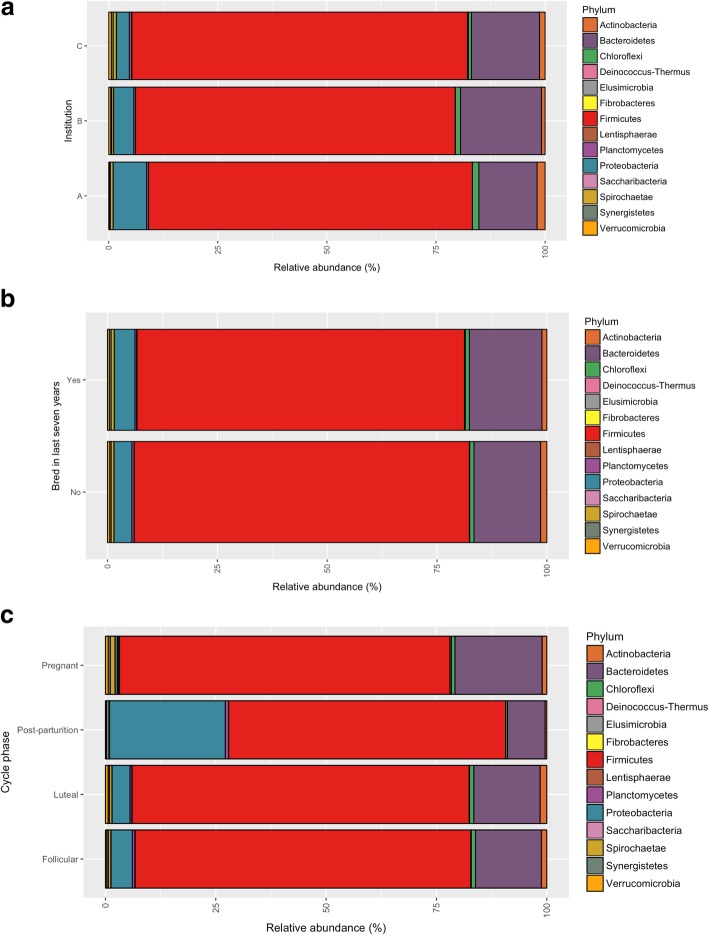
Relative abundance of bacterial phyla association with black rhino according to **a** institution, **b** reproductive success and **c** cycle phase

ID (*R*^2^ = 0.185, *F*_15, 111_ = 2.374, *p* < 0.001), institution (*R*^2^ = 0.048, *F*_2, 111_ = 3.675, *p* < 0.001), reproductive success (*R*^2^ = 0.016, *F*_2, 111_ = 2.470, *p* < 0.001) and ovarian cycle phase (*R*^*2*^ = 0.028, *F*_3, 111_ = 1.429, *p* = 0.008) were all significant predictors of microbiome composition (Fig. 1a–d). ID accounted for the most variation (18.5%) followed by institution (4.8%), ovarian cycle phase (2.8%) and finally, reproductive success (1.6%). At the phyla level, there were subtle differences in bacterial community composition between the three institutions (Fig. 2a) and between females that have bred in the last 7 years and those that have not (Fig. 2b). However, there were considerable differences in the relative abundance of bacterial phyla in samples collected post-parturition (*n* = 2) and during pregnancy (*n* = 19) compared with those collected during the luteal (*n* = 63) and follicular phases (*n* = 46), which were more similar to one another (Fig. 2c).

Indicator analysis identified seven genera (from a possible 253) with significantly different abundance in rhino gut microbiomes according to breeding success: five that were more abundant in breeding rhino and two that were more so in non-breeding rhino (Table 1). All of these genera except *Kurthia* (associated with non-breeding rhino) had lower relative abundance than the average relative abundance across all samples (mean average across all genera = 0.39%; S.E. = ± 0.080, range = < 0.001 to 9.17%) and thus represented rare microbial genera (Table 1). An additional indicator analysis identified 22 genera associated with ovarian status: one associated with the luteal phase, two associated with the luteal and follicular, five associated with pregnancy, seven associated with post-parturition and three associated with pregnancy and post-parturition, plus an additional four genera associated with the luteal phase plus a combination of one or two other phases (Table 2). Four of the five genera associated with breeding individuals (*Aerococcaceae*, *Atopostipes*, *Carnobacteriaceae* and *Solobacterium*) were also associated with pregnancy, post-parturition or both (Table 2). All genera associated with one or more particular phases of the ovarian cycle had lower-than-average relative abundance (Table 2).

**Table 1:** Results of indicator analysis showing bacterial genera with significantly greater prevalence in breeding or non-breeding black rhinos. The average relative abundance of each genera across all rhinos, along with the percent variation with faecal progestagen (fPMC) and faecal glucocorticoid metabolite concentrations (fGMC), is provided for context

Breeding status	Bacterial genus	Indicator analysis test statistic	*p* value	Average relative abundance (% ± S.E.)	% relationship with fPMC	% relationship with fGMC
Breeding	*Aerococcaceae*	0.289	0.050	0.01 (± < 0.01)	21.8	8.2
Breeding	*Anaerostipes*	0.257	0.045	0.02 (± 0.01)	9.8	0.2
Breeding	*Atopostipes*	0.398	0.020	0.03 (± 0.01)	18.1	1.2
Breeding	*Carnobacteriaceae*	0.370	0.020	0.01 (± < 0.01)	20.4	1.3
Breeding	*Solobacterium*	0.471	0.005	0.01 (± < 0.01)	28.2	10.2
Non-breeding	*Kurthia*	0.559	0.040	1.45 (± 0.47)	− 2.3	− 7.8
Non-breeding	*Rikenellaceae*	0.500	0.050	0.04 (± 0.01)	− 2.4	− 0.7

**Table 2:** Indicator analysis results identifying bacterial genera associated with different cycle phases, pregnancy and post-parturition. The average relative abundance of each genera across all rhinos, along with the percent variation with faecal progestagen (fPMC) and faecal glucocorticoid metabolite concentrations (fGMC), are provided for context

Indicative phase	Bacterial genus	Indicator analysis test statistic	*p* value	Average relative abundance (% ± S.E.)	% relationship with fPMC	% relationship with fGMC
Luteal	*Erysipelotrichaceae UCG-007*	0.474	0.040	0.02 (± < 0.01)	-3.1	-4.5
Luteal and follicular	*Lachnospiraceae NC2004 group*	0.785	0.005	0.06 (± < 0.01)	-13.8	-8.0
Luteal and follicular	*Rickettsiales incertae sedis genus*	0.663	0.005	0.10 (± 0.02)	-22.7	-14.8
Luteal and pregnant	*Clostridiaceae genus*	0.399	0.030	0.03 (± < 0.01)	16.9	11.5
Luteal and pregnant	*Ruminococcaceae V9D2013 group*	0.532	0.030	0.04 (± < 0.01)	18.2	-6.8
Luteal and post-parturition	*Carnobacterium*	0.415	0.025	0.23 (± 0.09)	8.2	-14.2
Luteal, follicular and post-parturition	*Lachnospiraceae ND3007 group*	0.686	0.010	0.07 (± 0.01)	-13.2	-6.9
Pregnant	*Aerococcaceae genus*	0.397	0.020	0.01 (± < 0.01)	21.8	8.2
Pregnant	*Lachnospiraceae NK3A20 group*	0.381	0.010	0.01 (± < 0.01)	14.3	-2.9
Pregnant	*Spirochaetaceae genus*	0.330	0.045	0.02 (± < 0.01)	16.6	13.7
Pregnant	*Sporobacter*	0.614	0.005	0.05 (± 0.01)	26.4	-10.4
Pregnant	*Succiniclasticum*	0.447	0.020	0.05 (± 0.03)	25.0	17.3
Post-parturition	*Aerosphaera*	0.595	0.040	0.01 (± < 0.01)	-4.5	-13.5
Post-parturition	*Atopostipes*	0.653	0.005	0.03 (± 0.01)	18.1	1.2
Post-parturition	*Bacillus*	0.534	0.005	0.01 (± < 0.01)	2.5	-4.7
Post-parturition	*Planococcaceae genus*	0.359	0.045	0.01 (± < 0.01)	2.5	3.9
Post-parturition	*Rummeliibacillus*	0.490	0.040	0.02 (± < 0.01)	-3.6	-6.9
Post-parturition	*Solibacillus*	0.568	0.040	0.10 (± 0.07)	1.0	2.1
Post-parturition	*Viridibacillus*	0.707	0.040	0.01 (± < 0.01)	6.8	2.0
Pregnant and post-parturition	*Carnobacteriaceae genus*	0.436	0.015	0.01 (± < 0.01)	20.4	1.3
Pregnant and post-parturition	*Erysipelotrichaceae UCG-004*	0.391	0.015	0.01 (± < 0.01)	19.2	24.3
Pregnant and post-parturition	*Solobacterium*	0.458	0.015	0.01 (± < 0.01)	28.2	10.2

### Relationships between microbiome composition and faecal hormone metabolite concentrations

**Fig. 3:**
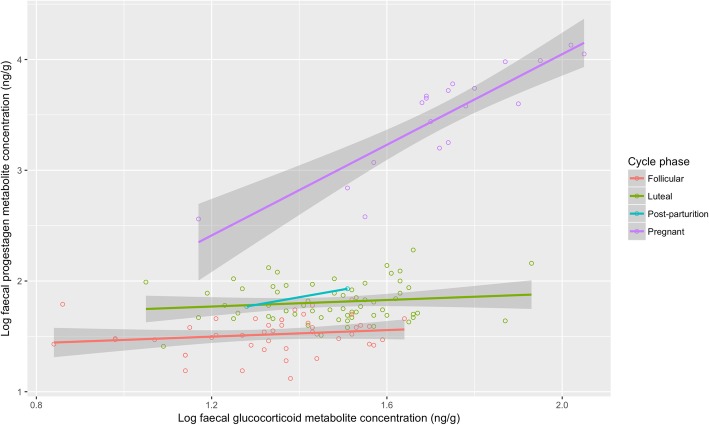
Relationship between log faecal progestagen and log faecal glucocorticoid metabolite concentrations according to the two cycle phases, pregnancy, and post-parturition

There was a statistically significant positive correlation between log progestagen and log glucocorticoid faecal metabolite concentrations (*r* = 0. 628, *p* < 0.001; Fig. 3). Ovarian cycle phase (*X*^2^ = 375.4, *p* < 0.001) but not breeding success (*X*^2^ = 0.1, *p* = 0.782) had a significant effect on log progestagen faecal metabolite concentrations (Fig. 3). Similarly, ovarian cycle phase (*X*^2^ = 32.6, *p* < 0.001) but not breeding success (*X*^2^ = 0.5, *p* = 0.502) had a significant effect on log glucocorticoid faecal metabolite concentrations (Fig. 3). In both cases, samples associated with pregnancy showed higher metabolite concentrations (Fig. 3).

**Fig. 4:**
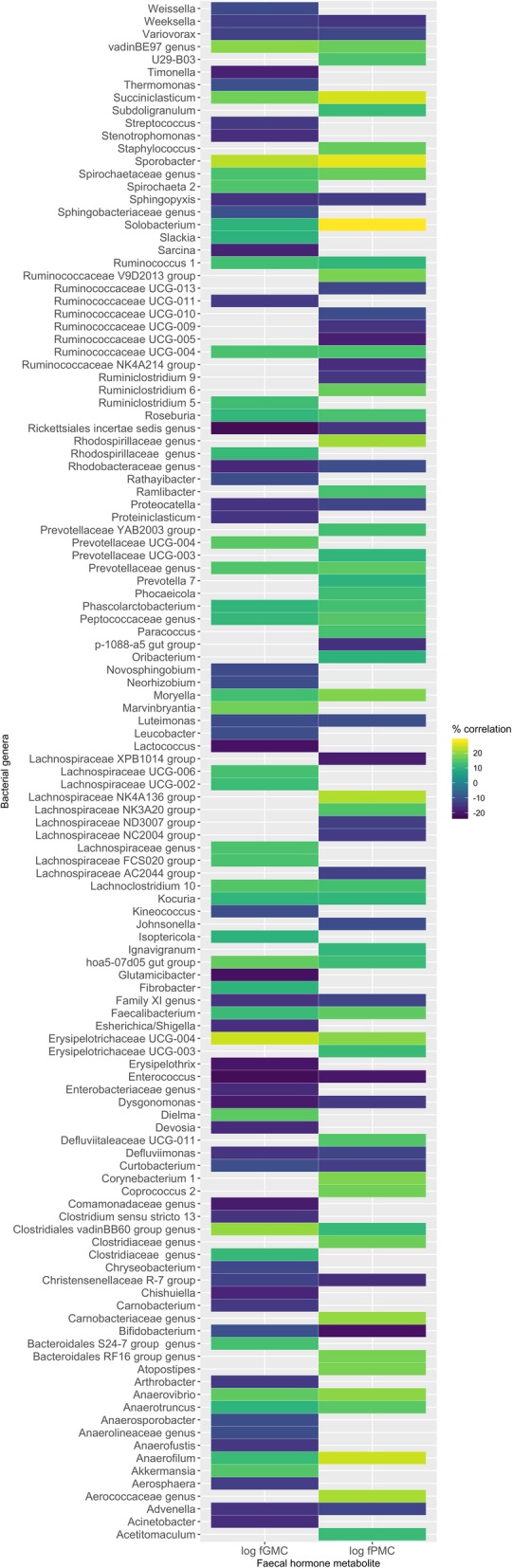
Heatmap showing correlation coefficient between relative abundance of bacterial genera and faecal hormone metabolite measures. Only genera with coefficient > ± 0.10 for one or both hormones are shown (a blank tile indicates this genus did not have a prevalence of > ± 0.10 for a given hormone)

Of 253 bacterial genera, we identified 74 (29.2% of genera) with correlation of greater than ± 10.0% for log faecal progestagen metabolite concentrations (47 positive and 27 negative relationships) and 83 genera with correlation greater than ± 10% (32.8% of genera) for log faecal glucocorticoid metabolite concentrations (37 positive and 46 negative relationships), although these were not statistically significant (all *p* > 0.05; Fig. 4 and Additional file 1: Figure S2). Of these, nine had greater than ± 20.0% correlation for faecal progestagen metabolite concentrations (eight positive and one negative) and seven were greater than ± 20% for faecal glucocorticoid metabolite concentrations (three positive and four negative) (Fig. 4). Generally speaking, if a microbial genus had a positive correlation with one hormone, the relationship was also positive with the other hormone, and vice versa (Fig. 4 and Additional file 1: Figure S2). Nine of the 15 genera associated with pregnancy and/or post-parturition had a positive (± 10%) relationship with log faecal progesterone metabolite concentration, but their relationships with log faecal glucocorticoid metabolite concentrations were more variable (Table 2). However, four genera (*Aerococcaceae*, *Atopostipes*, *Carnobacteriaceae* and *Solobacterium*) that were significantly associated with breeding rhino and pregnancy and/or post-parturition (Tables 1 and 2) also had a positive relationship (> ± 10%) with faecal progestagen metabolite concentrations (Fig. 4). Two of these (*Aerococcaceae* and *Solobacterium*) also had a positive relationship (> + 0.10) with faecal glucocorticoid metabolite concentrations (Table 1; Fig. 4).

## Discussion

Relationships between the gut microbiome and hormone production are increasingly well-characterised in humans and model organisms, although less so in non-model organisms, particularly those of high conservation concern. Here we show that the microbiome of captive black rhino is significantly associated with ID, institution, reproductive success and ovarian cycle phase. In addition, a number of bacterial genera are linked to higher faecal hormone metabolite concentrations, reproductive success, and ovarian cycle phase.

We show that progestagen and glucocorticoid metabolites are correlated in black rhino, and find evidence that both glucocorticoids and progestagens are higher during the luteal phase than the follicular phase, and both are much higher during pregnancy. Hormone production is known to vary temporally for host organisms; for example, parous Asian elephants exhibit cyclic changes in glucocorticoids, peaking during the follicular phase, and faecal oestrogen and glucocorticoid metabolites are positively correlated in female giant panda (*Ailuropoda melanoleuca*) [34]. We also provide novel evidence that gut microbial communities change concurrently across the different phases of the ovarian cycle in black rhino; in particular, the gut microbiome during pregnancy and post-parturition are significantly different. These changes in microbiome composition appear to be, in part, linked to differences in hormone concentration. We show that nearly a third of all bacterial genera have a relationship, albeit weak, with either faecal progestagen or glucocorticoid metabolite concentrations, indicating numerous complex interactions between members of the host microbiome and associated host hormone profiles. Such interactions, in addition to host and environmental influences, can have considerable implications for the metabolite profiles of microbiomes and subsequent host physiology [56, 57]. Thus, although we also find specific members of the microbial community linked to multiple measures of reproductive success, the composition of a considerable portion of the entire microbiome appears to interact with downstream hormone production and reproductive success [58]. Although these patterns are informative, we are not able to identify the directionality between microbiome composition and reproductive traits (including breeding success, pregnancy and hormone concentrations) and thus, it is not clear whether microbiome composition is affected by the physiological changes associated with pregnancy or whether the differences in microbiome composition are a driver of breeding success. Further work is required to understand how these complex microbial communities work together to influence hormone production and reproductive success, as well as the implications of these for microbial profiles and subsequently other fitness traits in hosts. Moreover, because faecal samples can be non-invasively sampled, there is the potential for using the composition of the gut microbiome as a biomarker for both gut and reproductive health in captive and wild animals.

Four bacterial genera in particular (*Aerococcaceae*, *Atopostipes*, *Carnobacteriaceae* and *Solobacterium*) were associated with breeding success, pregnancy, and higher hormone metabolite concentrations (particularly progestagen), suggesting these groups are linked to reproductive output. Similarly, Vlčková et al. [23] showed *Clostridium cluster XIVb*, *Oscillibacter* and genera from *Anaerolineaceae* were associated with higher levels of faecal glucocorticoid metabolite concentrations in *G. g. gorilla*. Although keystone microbes have been identified in other systems [48, 59], the role of these microbial groups in driving hormone production and/or reproductive success is unclear. More work is required to determine whether particular microbes associated with breeding success can be used to improve reproductive output of captive black rhino, either directly through probiotic administration or indirectly through diet manipulation and/or prebiotics. Given that captive rhino have significantly altered microbiomes in comparison to wild individuals [60], there may also be value in identifying microbial taxa associated with wild counterparts, particularly those exhibiting high reproductive output, which may be used to improve the health and fitness of captive rhino.

It has previously been demonstrated that various components of the maternal gut and vaginal microbiomes alter throughout gestation and lactation in humans, including increases in *Proteobacteria* and *Actinobacteria* [61–63]. However, we actually saw decreases in both of these phyla for pregnant rhino, with concurrent increases in *Synergistetes*, *Spirochaetae* and *Verucomicrobia*. Post-parturition, these were replaced by substantial increases in Proteobacteria. Changes in the relative abundance of gut microbial groups may also be an artefact of other physiological factors associated with the oestrous cycle and pregnancy, such as differences in metabolism [61]. Another potential driver of these microbial changes may be oxytocin, which influences gastric motility [64, 65] and thus likely affects the gut microbiome. Interestingly, the production of oxytocin is also linked to microbiome composition [66, 67]. Therefore, the gut microbiome of recently calved rhinos may well be altered as a result of high oxytocin production during birth and lactation. There is also evidence that maternal gut microbiota can transfer to the gut of offspring via mothers milk [68] and differences in the gut microbiome of mothers corresponds to differences in milk microbiome and offspring gut microbiomes [69]. Therefore, changes in maternal gut microbiome may also be adaptive to ensure their offspring are seeded with the correct microbiota during early development. Thus, it would be interesting to identify links between the gut and milk microbiome composition of mothers and their offspring, particularly given that maternal gut microbiome composition can have implications for offspring immunity [69].

Indeed, interactions between host diet, microbiome composition and physiological profiles of black rhino, and the implications of these for reproductive success, are a key area for further work. Poor diet of other rhino species in captivity has been linked to low reproductive output as a result of high estrogenicity (phytoestrogens) in the feed that block oestrogen receptors [70], which may be linked to the concentration of microbially derived metabolites [71]. Similarly, differences in circulating leptin and insulin concentrations have been linked to acyclicity in oestrous cycles of horses as a result of obesity, which may be linked to gut microbiome structure [72, 73]. We did not characterise the diet of individual rhinos or institutions, although we did identify significant differences in gut microbiome composition of black rhino according to institution, which may arise from differences in diet and husbandry [74–77]. However, despite statistical significance, there is surprisingly little variation in microbiome composition between institutions (4.8%), suggesting host institution does not have too large an impact on host microbiome. That said, we also show there is considerable within-individual variation in the gut microbiome of black rhinos (18.5%), indicating the microbiome is temporally dynamic which is, in part, driven by variation in physiological factors such as endocrinology. However, rhino ID, institution, breeding success and cycle phase all together only accounted for 27.7% of the variation in gut microbiome composition, and thus, other environmental and host factors including diet, age, social interactions and host genotype are also likely influencing the composition and function of these complex communities [22, 41, 78–81]. Variation between individuals in gut composition, and factors that influence this, may affect the success of prebiotic and probiotic strategies, and further work is required to determine the feasibility and efficacy of this type of approach.

## Conclusions

We identified significant differences in black rhino gut microbiome composition according to ID, institution, breeding success and ovarian cycle phase. In particular, the gut microbiome during pregnancy and post-parturition was significantly altered. Around a third of bacterial genera showed more than ± 10% correlation with either progestagen and/or glucocorticoid concentration. Twenty-four out of 25 genera indicative of a particular phase of the ovarian cycle and/or breeding success had a lower-than-average relative abundance in the gut microbiome, and thus, the rare microbiota appears to be related to reproductive output in black rhino. Indicator analysis identified four genera (*Aerococcaceae*, *Atopostipes*, *Carnobacteriaceae* and *Solobacterium*) that were significantly associated with breeding success, pregnancy and higher hormone concentrations. There is potential to develop these into probiotics to improve the breeding success of black rhino in zoo-based conservation breeding programmes. Further work is required to understand the efficacy and feasibility of this, either directly through microbial augmentation (probiotics) or indirectly via dietary manipulation or prebiotics, as well as wider interactions between diet, gut microbiome and host physiology.

## Additional files

Additional file 1: **Table S1.** Information on rhino institution, age and breeding status for individuals included in the study. **Figure S1.** Rarefaction curves for each sample according to observed richness and Shannon diversity measures. **Figure S2.** Relationship between bacterial genera and faecal progestagen (fPMC) and faecal glucocorticoid metabolite concentrations (fGMC) across all individuals. (PDF 500 kb)

Additional file 2: R Markdown containing all analysis code. (RMD 21 kb)
