# Supplementary material for: Rare gut microbiota associated with breeding success, hormone metabolites and ovarian cycle phase in the critically endangered eastern black rhino
Source: Microbiome. 2019 Feb 15;7:27. doi: 10.1186/s40168-019-0639-0 (PMC6377766; doi:10.1186/s40168-019-0639-0)
Supplement: Supplementary file 1 — Table S1. Information on rhino institution, age and breeding status for individuals included in the study. Figure S1. Rarefaction curves for each sample according to observed richness and Shannon diversity measures. Figure S2. Relationship between bacterial genera and faecal progestagen (fPMC) and faecal glucocorticoid metabolite concentrations (fGMC) across all individuals. (PDF 500 kb) [file 40168_2019_639_MOESM1_ESM.pdf]

Supplementary Material – Antwis et al. Rare microbiota associated with breeding success, hormone production and ovarian cycle phase in the critically endangered eastern black rhino

Table S1

Information on rhino institution, age and breeding status for individuals included in the study.

ID	Institution	Age	Breeding individual?
Rhino 01	C	27	No
Rhino 02	A	8	Yes
Rhino 03	C	18	Yes
Rhino 04	C	18	No
Rhino 05	A	13	Yes
Rhino 06	A	5	No
Rhino 07	A	12	No
Rhino 08	C	21	No
Rhino 09	C	7	No
Rhino 10	A	22	No
Rhino 11	C	14	Yes
Rhino 12	B	11	No
Rhino 13	C	40	No
Rhino 14	B	10	No
Rhino 15	C	9	No
Rhino 16	C	19	Yes

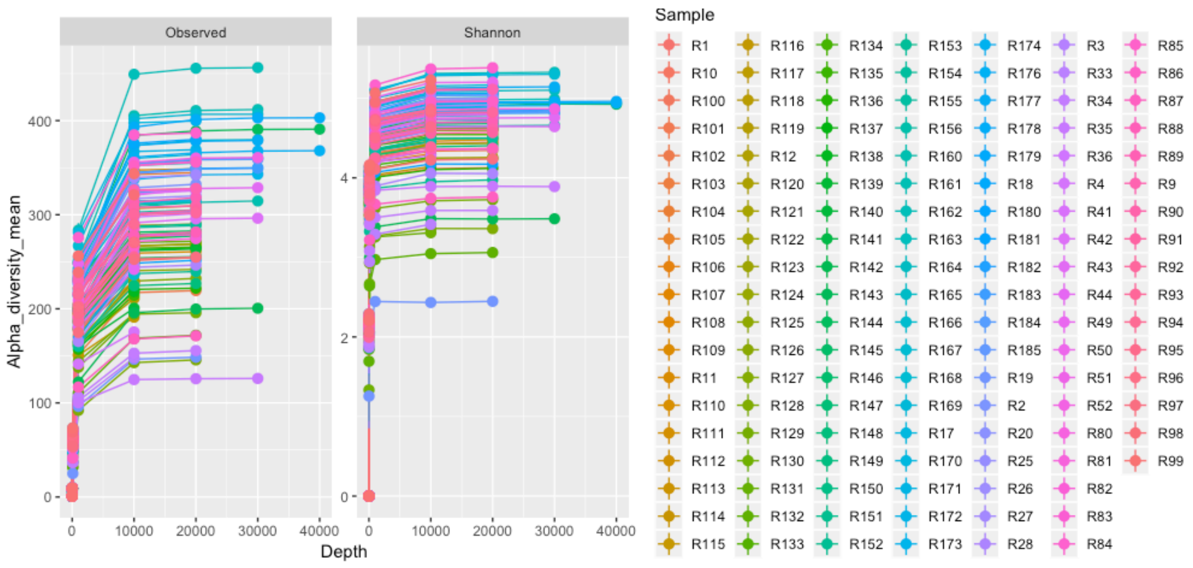

Figure S1

Rarefaction curves for each sample according to observed richness and Shannon diversity measures.

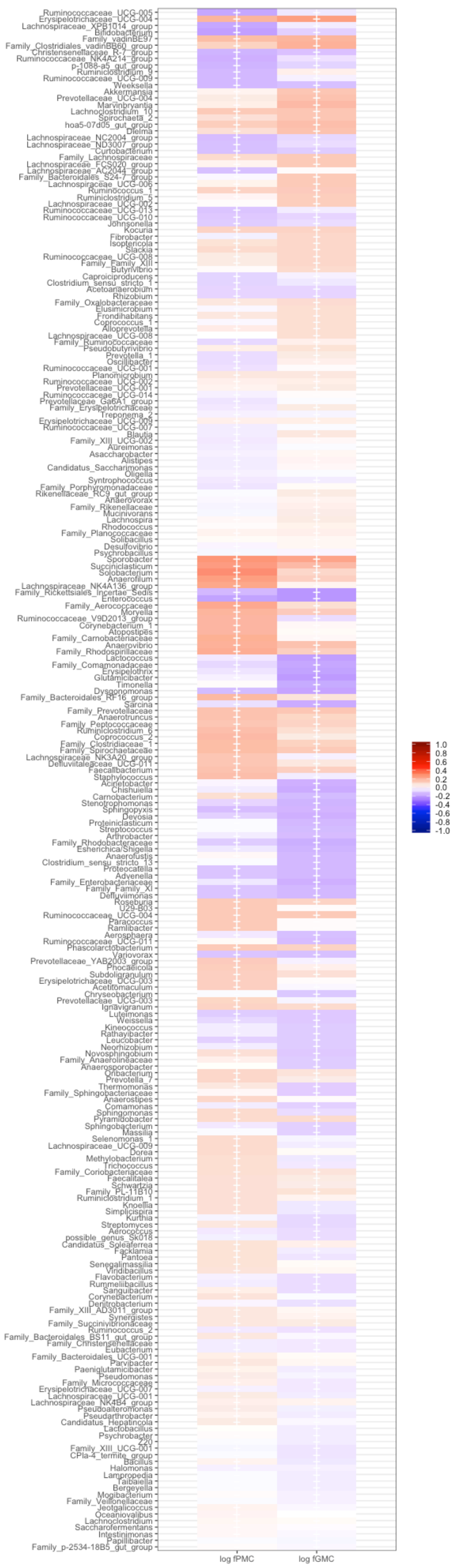

Figure S2

Relationship between bacterial genera and faecal progestagen (fPMC) and faecal glucocorticoid metabolite concentrations (fGMC) across all individuals.
